# How can specialist investigation agencies inform system-wide learning for patient safety? A qualitative study of perspectives on the early years of the English Healthcare Safety Investigation Branch

**DOI:** 10.1177/13558196241291816

**Published:** 2024-11-05

**Authors:** Amanda Crompton, Justin Waring, Carl Macrae, Charlotte Overton, Rosie Benneyworth

**Affiliations:** 1 Associate Professor in Public Policy and Management, Centre for Health Innovation, Leadership and Learning, Nottingham University Business School, 6123 University of Nottingham, Nottingham, UK; 2 Professor of Medical Sociology and Healthcare Organisation, Health Services Management Centre, 1724 University of Birmingham, Nottingham, UK; 3 Professor of Organisational Behaviour and Psychology, Centre for Health Innovation, Leadership and Learning, Nottingham University Business School, 6123 University of Nottingham, Nottingham, UK; 4 Research Fellow, Yorkshire and Humber Patient Safety Research Collaboration, UK; 5 Chief Executive Officer, 700216 Healthcare Services Safety Investigation Branch, UK

**Keywords:** investigation, patient safety, learning, safety investigation, discipline: social science, qualitative methods, evaluation

## Abstract

**Objectives:**

System-wide learning for patient safety is a core challenge for the health care sector, despite the prevalence of localised reporting and learning approaches. There is growing interest in how health care services could emulate other safety-critical sectors with the introduction of specialist safety investigation agencies to inform sector-wide safety. This paper reports on a study of the introduction and early operation of one such agency in the English health and care system.

**Methods:**

This was a qualitative interview study carried out between 2019 and 2021 and co-designed through a partnership between University researchers and the Executive Team from the Healthcare Safety Investigation Branch (HSIB) to explore the organisational development of this ‘first of type’ organisation. The study involved interviews with 33 internal and external stakeholders and documentary analysis of HSIB reports.

**Results:**

The study findings highlight the organisational competencies and developmental challenges experienced in the early years of HSIB operations focusing on (i) independence and fit within the wider system; (ii) the selection and scope of investigations; (iii) the methodology and investigation approach; and (iv) the skill and competencies of investigators.

**Conclusions:**

This study offers practical learning to health care decision-makers about the importance of securing independence and integration, about the production of system-wide learning, the standardisation of robust methodologies and the support for a multidisciplinary specialist workforce.

## Introduction

System-wide learning for patient safety remains elusive. Existing methods that could inform such learning tend to be partial, uncoordinated and designed to serve purposes other than learning. For example, incident reporting systems tend to focus on contributory factors at the local level rather than the wider care system.^1,2^ Public or government inquiries into major safety events can provide far-reaching recommendations, but are costly, time-consuming and impractical as a model of routine learning.^3,4^ Regulators set and assure the standards of care, but their role is typically focused on organisational compliance rather than system-wide improvement.^3^ Notwithstanding the range of agencies involved in regulating patient safety,^5^ there remains a need for a more systemic approach to population-level learning for patient safety that utilises existing surveillance systems to identify significant trigger events that can inform lessons and recommendations.^6^

In other safety critical sectors, independent investigations are routinely conducted in response to serious operational failures and safety breaches to determine contributory factors, establish accountabilities and make recommendations for sector-wide improvements. Learning from these sectors, Macrae and Vincent^7,8^ have advocated for the introduction of specialist safety investigation agencies in the health care sector to carry out independent investigations into system-wide patient safety issues and to deliver practical recommendations for system-wide improvement. The Healthcare Safety Investigation Branch (HSIB) was established in 2016 in the National Health Services (NHS) based on recommendations from an Independent Expert Advisory Group,^9^ and the National Investigation Board for Health and Care Services (NIBHC) in Norway provides another example of this type of agency.^10^

While the HSIB may be considered a ‘first of type’ health care safety investigatory body, there is much to learn about its ability to affect system-wide learning from the experiences of pre-existing health care inspection and regulatory agencies. Research highlights questions about the accuracy and reliability of investigation methods, the skills of investigators, and the scheduling of inspections.^3,11^ For example, the position and integration of inspection agencies within the wider governance framework can impact the ability to inform decision-making and influence change.^3^ Furthermore, the reactive and problem-focused character of investigations means they are not always orientated towards system-wide learning and improvements.^11^ Although HSIB is not a regulatory or an improvement agency, it does bear similarities with such bodies by seeking to produce recommendations that are aimed at system-wide improvement.

Research on safety investigation agencies in other sectors also suggests that these bodies can face a number of developmental and operational challenges.^12^ First, investigations are widely presented as being independent, but many agencies can have close governmental or sectoral ties. Consideration is thus needed of the extent of organisational, financial and legal freedoms and the implications of this for transparency, objectivity and credibility. Second, investigations can vary in scope, from case-based to sector-wide, with implications for the depth of specialist insight and the breadth of system-wide learning. Third, there is a lack of standardisation in investigation methodologies both within and between sectors, which can compromise the rigour of the investigation process, validity of recommendations and potential for system-wide learning. This raises questions about the real, or perceived, investigatory expertise of such agencies. Fourth, investigations require the involvement of different specialists, especially those qualified in investigation methodologies, but the precise configuration of these competencies and skills is under-specified. Health care safety investigation agencies such as the HSIB in England and the NIBHC in Norway are likely facing similar challenges.^8,13^

The study reported in this paper examined the developmental challenges experienced in the early years of HSIB (2017–2021). At the time of this study, HSIB was hosted by NHS England, the national executive agency for the English NHS. HSIB operated two separate programmes. ‘National’ investigations focused on safety concerns that represented a systemic risk across the wider NHS, often triggered in response to reported incidents or local investigations within the NHS. ‘Maternity’ investigations focused on all reported safety incidents in maternity services across England, thereby replacing local in-house investigations. The two programmes were organised separately, and in this study we report on the national investigation programme only.

Since the completion of this study, there have been significant changes in the governance and organisation of HSIB. A review of HSIB’s leadership and culture^14^ highlighted numerous challenges including disunity between organisational goals and professional groups which, to some extent, complicated the design and conduct of investigations. In October 2023, HSIB was re-branded and re-launched as the Health Services Safety Investigation Body (HSSIB), a fully independent public agency with a remit to investigate patient safety concerns across the NHS and in private health care settings in England where safety learning could improve NHS care.^15^

## Methods

We report on the findings of a qualitative study on the introduction and early development of HSIB from the perspective of internal and external stakeholders. It was carried out between June 2019 and February 2021. The study was co-designed with senior leaders from HSIB between 2018 and 2019, with a focus on the organisational competencies and developmental challenges experienced in its early years. This involved three in-person and virtual roundtable meetings to clarify the learning needs and the study focus. The study was given favourable ethical review by Nottingham University Business School, University of Nottingham Ethics Committee (Reference 201819032).

### Sampling and recruitment

HSIB staff were sampled purposively on the basis of their involvement in different aspects of HSIB’s governance and operational processes, including executive leadership, investigation, analytics, communications and administration. Following the co-design activities, information about the study was circulated to all staff and all participants were recruited via email by the research team. The identity of all participants remained undisclosed to ensure confidentiality and anonymity.

External stakeholders were sampled purposively on the basis of their roles with other agencies in the wider health care system. External stakeholders included, for example, NHS agencies, health care and professional regulatory bodies, charitable and advocacy groups, and specialist research groups. They also included representatives from NHS organisations that had participated in or been the subject to an HSIB investigation. Participants were identified and recruited via email by the study team, without consultation with HSIB.

In total, 33 people participated in the study, including 19 members of HSIB staff of whom six took part in follow-up interviews 9–12 months after the first interview, and 14 external stakeholders (Table 1). Given the size of the sample relative to the workforce of HSIB during the study period, it was recommended by research governance advisors and HSIB leaders not to ascribe occupational roles to extracts of data to ensure the confidentiality of participants.

**Table 1.** Study participants.

Participant group	Number interviewed
Senior leaders	2
National investigators	5
Principal investigators for the first six reports	2
Members of the Intelligence Unit	7
Administrators and support staff	3
External stakeholders from the following organisations • Care Quality Commission (1) • National Institute for Health and Care Excellence (1) • NHS Improvement (3) • Royal colleges (3) • The National Joint Registry (4) • The National Patient Safety Agency (1) • Trust level patient safety and risk management lead (1)	14
Total	33

### Data collection and analysis

The study used semi-structured qualitative interviews. All interviews followed a topic guide that investigated participants’ views about: (a) HSIB’s purpose, organisation and governance; (b) HSIB’s investigation processes; (c) perceptions about the impact of HSIB on the wider health system; and (d) recommendations for improvements in HSIBs working practice. The topic guide was used dynamically to explore particular topics with different participants.^16^ For example, HSIB staff were asked to reflect upon their experience of working in HSIB, their role in investigation and dissemination processes, and the quality of reporting. For external stakeholders, questions focused on their perceptions of the investigation process, the quality of investigations and reports, the contributions to patient safety improvement, and HSIB’s position in the health care system. Interviews were carried out by AC, JW and CO and ranged from 40 to 90 min. Five interviews were carried out in person and 28 were conducted by telephone. With participant consent, 31 interviews were digitally recorded for the purposes of transcribing and analysis. Two participants (HSIB staff) did not consent to having their interview recorded due to concerns about anonymity.

Qualitative data analysis involved stages of interpretative inductive coding, constant comparision and thematic analysis of transcribed interviews.^17–19^ First, three members of the research team carried out independent review of five transcripts each (15 transcripts in total) to identify relevant lines of analysis. Identified lines of interpretation were then reviewed and deliberated as a group to agree a coding approach. Transcribed data was then subject to systematic coding with sections of text tagged and extracted into a table, together with interpretative comments and coding labels. Extracted data were reviewed following a constant comparison approach to ensure descriptive accuracy and internal consistency.^18^ Through the process of axial coding, first order extracts were grouped into second order categories based upon similarity, which were then related back to the existing literature on the challenges faced by other investigation bodies to arrive at four overarching themes.^10,12^ For example, HSIB staff talked about their experiences of the investigation processes, highlighting different approaches to leading and designing investigations, their familiarity with and preferences for investigation methods, the involvement of different people, and experiences of carrying out investigations. We labelled these with annotated notes and categorised them as ‘Methodologies’, ‘Planning’, ‘Rigour’ and ‘Standardisation’. Categories were then summarisd under the theme ‘Investigation method and approach’.

## Results

In this section we explore our four overarching themes. We begin by exploring perceptives of independence and HSIB’s fit within the wider system. We then examine HSIB’s investigatory process in terms of the selection, scope and focus of their investigations and the investigation methods and approach. Finally, we discuss the significance of HSIB’s multidisciplinary expertise and skills.

### Independence and fit within the wider system

A key challenge for HSIB was to establish itself as sufficiently independent within the health care governance landscape. This was seen as a difficulty with HSIB being both part of the wider NHS, and yet simultaneously distinct from it. This tension was important as HSIB required independence in what and how it investigated, while drawing on system-wide connections to influence the service following its investigations. With reference to the independence of its investigations, HSIB executives talked about basing their approach on the principles of ‘*safe space*’, a ‘*no blame*’ culture and, ultimately, as being different from other regulatory bodies. Similarly, HSIB investigators stressed the importance of being independent from bodies such as the Care Quality Commission (CQC; independent regulator of health and social care in England) and NHS England, which contributed to the perception of it being ‘*trustworthy*’ and ‘*approachable*’. They also described the importance of focusing their work on ‘*systemic problems with the NHS*’ (Internal stakeholder 5), which further demonstrated its unique function.

*I think when you speak to the staff when you’re doing interviews and you tell them that you’re not a regulator, it actually puts them at ease. It gives us a different stance. We’re approaching it from a different angle. They can tell that very quickly from the interviews in the style of question that we ask because our questions are generally all about the system that they’re working in. And they can tell very quickly that we’re asking questions in a different way. *(Internal stakeholder 9)

External stakeholders reflected positively on HSIB’s perceived independence and goal of offering a *‘safe space’* for enquiry and learning.

*I think HSIB having the safe space is the right thing but actually that should extend and if the government is really interested in patient safety then these should be very much about learning and driving improvements and it shouldn’t … yeah, I think that should be extended to any serious incident investigation.* (External Stakeholder 2)

*[T]hey’re clearly a very important source of information and insight for us in relation to the kinds of underlying contributory factors and causes that sit behind some patient safety concerns and incidents. And it’s that kind of insight that we can then use to either do national work or support local work, to tackle the causes of things going wrong.* (External stakeholder 12)

However, some external stakeholders were unclear about the specific contribution of HSIB to the existing improvement infrastructure, suggesting that it was potentially decoupled from other initiatives.

*…my overall view is that it’s doing the right things but it needs to be connected to the engine that drives improvements. And that engine at the moment is rather ill-defined.* (External stakeholder 3)

At the time of the study there was no clear responsibility regarding the uptake or monitoring of HSIB recommendations, further illustrating a sense of de-coupling from the wider health care system.

*…it might not be HSIB that is responsible for doing that but someone needs to say well come on guys, why haven’t you learnt from the last investigation? And why haven’t you actually put in place the recommendations that were made by HSIB? So for me that’s a real weakness of the system. *(External stakeholder 4)

The overall impression from external stakeholders was that HSIB’s influence was compromised by its lack of authority and direct influence within the system, which was reflected in the view that ‘*only organisations with* “*teeth*” *can make recommendations stick*’.

*[T]hey’re independent from the government and they give recommendations at the end… currently they don’t have any political trounce to make them happen.* (External stakeholder 3)

### The selection, scope and focus of investigations

HSIB investigations were often described as producing system-wide learning. This learning was based on the application of Human Factors expertise,^20^ which is commonly used in other safety-critical sectors. It aims to identify latent factors located in the wider system that, once addressed, will inform improvements across multiple elements of the care system. In practice, this involved learning from a *‘reference’* or *‘trigger’* event that had been reported within the service or by service users, or identified by HSIB’s Intelligence Unit. However, those carrying out investigations talked of ambiguities in the selection of reference events and whether they necessarily represented a common system issue.

*I think we want to be a bit more systematic about it and being able to explain a bit more why we do something when we do it…. Rather than saying we decided to investigate this particular event because this event happened to be particularly interesting or serious or whatever it happened to be. *(Internal stakeholder 7)

Recognition of this led to HSIB’s Intelligence Unit having a more prominent role in gathering and analysing relevant data sources to scope out and confirm the significance of an event. It further brought about the introduction of a new Scrutiny Panel, in which clinical and safety specialists helped to define the purpose and focus of investigations.

*[T]he Scrutiny Panel has kind of two parts to it now. So, we have the responsive part, where we kind of will present any [incident] related to recent referrals that we think we can potentially investigate. … And then there’s the second part which is kind of the focus area part, which is where we think we should be.* (Internal stakeholder 4)

Investigators also talked about difficulties in extrapolating system-wide learning from a given reference event. Some described investigations as a ‘*deep-dive*’ into a specific event that could potentially risk replicating the incident investigation already carried out by a given NHS hospital. Others talked about the lack of an agreed systematic analytical method for developing more general or system-wide learning from individual reference events. For some, the process of inferring system risk factors was highly variable and seemingly based on intuition and ‘*hunches*’.

*It’s just about moving from the sharp end to the blunt end. For me it’s not really so much about using a particular tool but from a sort of systems perspective because you’re talking about such a big picture, you’re having to use kind of abstract high-level ideas and apply them down. Because otherwise there’s no way of you sort of getting into your head how this massive system works or how things fit together*. (Internal stakeholder 6)

### Investigation methods and approach

Many of the reported uncertainties surrounding the scoping and selection of investigations, together with the challenges in extrapolating system-wide learning, were compounded by the absence of an agreed and standardised investigation method. HSIB staff talked of using a variety of investigation approaches, with some describing a type of ‘*tool-bag*’ approach in which different techniques were combined, often based on their past experiences in either health care or other industries.

*I think we’ve still got more experimenting to do with the different models. And how we use those. I think we’ll need to use a method in several different investigations before I think it will really be of use to other people. *(Internal stakeholder 7)

It was also described how investigations were carried out with little forward planning about desirable data sources, interview topics and participants, or analytical methods. For example, stakeholders from one reference event site reflected that the HSIB investigations did not appear to involve sustained or in-depth work.

*They just turned up and we’d organised a room and we all met with them in the room. And then we talked about what they would need and what they might want and you know, we shared any documents or any guidelines or policies that they asked for. But that was it. So, it was a question of emailing them a few documents. It was nothing more. *(External stakeholder 2)

The need for a more rigorous and standardised approach prompted the introduction of an Investigation Improvement Group to make recommendations about the effectiveness of various investigation methodologies.

*…through the Improvement Group we are running a piece of work to look at how we use tools and how we evaluate the use of those tools within our investigations…I haven’t really been able to find out from people that there’s any evidence that one tool is any better than another tool. And particularly in health care because most of them haven’t been used in health care before, a lot of them are derived from transport accident investigation.* (Internal stakeholder 1)

Some HSIB investigators still questioned the premise of a standardised investigation model, suggesting that if HSIB advocated a particular approach it would be vulnerable to criticism.

*I think it’s a very, very valid point, if we publish a particular safety analysis model, we will open ourselves to criticism that if we’d have used a different model we’d have got a different result. *(Internal stakeholder 8)

Variations and inconsistencies in the investigation approach, together with ambiguities about how to extrapolate from reference events to system-wide learning, therefore seemed to complicate the goal of generating learning.

### Multidisciplinary expertise and skills

The HSIB workforce was diverse in terms of professional backgrounds, experience in safety investigations, and understanding of the health care sector. This diversity was valued for enhancing the quality of the investigation processes by, for example, bringing together people with experience of frontline services and those with experience of safety investigations in other safety-critical sectors, especially those with advanced qualifications in Human Factors. However, this diversity was also seen to have led to operational challenges which, when coupled with the lack of a standardised investigation approach, could undermine the quality and consistency of HSIB’s work.

*I think there has to be an acknowledgement that in any new organisation it’s difficult and it can be … there is often a pull for certainty and a pull for what people are familiar with. And sometimes that’s what creates the behaviours that are unhelpful.* (Internal stakeholder 3)

One problem associated with workforce diversity related to the allocation of staff to investigations, which in the early period involved a *‘taxi rank’* system of queued availability rather than targeting expertise to a given safety issue. This meant that, sometimes, staff with relevant expertise were not utilised to lead investigations.

*… sometimes there are discussions before then, so you’ll get a heads-up that might say we’re going to take this case today. So just to let you know you’re next in line to be leader and you go fair enough, okay. Other times there are conversations that happen and then they don’t quite match what comes out of Scrutiny. *(Internal stakeholder 12)

The taxi rank system was rapidly replaced with an approach that matched safety issues and contexts to the relevant skills and experiences of staff, and also gave more consideration to combining investigators in terms of their experiences in and outside the health care sectors.

The diverse backgrounds of HSIB personnel was described as creating a culture clash between those drawn from health services and those from other sectors, where the former had deep contextual understanding and the latter more technical expertise in safety investigation methods, but often with a disregard to the complexities and specificities of health care.

*I think there is something about being a health care professional which is of value but it also may blinker you to other things. So, it’s valuable having non-health care professionals in the team as well… they can point out the ridiculous inconsistencies which the rest of us have just taken for granted.* (External stakeholder 8)

## Discussion

HSIB might be perceived as a ‘first of type’ health care safety investigation agency, which makes it an important source of learning for England and elsewhere. The study reported here makes an initial step in this endeavour by focusing on the early years of HSIB’s introduction, the development of its organisational competencies and its steps towards making contributions to system-wide learning. Informed by the wider literature, this study distils this learning against the principles of independence, scope and focus of investigations, investigation methodology and multidiscplinary expertise identified by Roed-Larson and Stoop.^12^ Through our study we make suggestions for how decision-makers in health care systems might deal with key challenges.

Independence is typically viewed as a founding principle of safety investigation bodies such as HSIB^12,13^; it provides the freedom to impartially identify and select appropriate areas of investigation, and should enable sufficient separation from the organisations that are investigated and to whom recommendations are issued. This study found there were no significant concerns regarding HSIB’s independence to select and carry out its national investigations. There were questions, however, about whether HSIB was sufficeintly integrated in the wider care system, and whether this would limit the translation and enactment of recommendations, or preclude follow-up and monitoring of responses. The field of patient safety governance is complex^5^ and at the time of our research, HSIB remained on the periphery of the ‘system of control’.^21^ Specifically, much of the responsibility for the translation of learning into practice remained with other agencies, such as NHS England, regulatory bodies or NHS care providers. As noted in the wider literature, the dilemma of independence stems from the need to be both part of, and apart from, the sector under investigation.^12^ This means that safety investigation agencies must give consideration to how independence can be maintained while also building influence and authority.^22^ In particular, investigation bodies need to pay close attention to developing a variety of channels through which learning can be initiated and enabled, including clear agreements on modes of communication, expectations for action planning, and follow-up review. This may involve establishing relatively formal networks or conduits of knowledge exchange^23^ and thinking about how learning can be communicated in ways that go beyond a final report to include diverse modes and media relevant to different stakeholder groups.^24^

The scope and focus of investigations is central to the effectiveness of an investigation body. We found a deeply expressed commitment for HSIB to conduct investigations that enabled learning across the wider system of NHS care. However, there were ambiguities about the focus of investigations, particularly regarding the initial selection of ‘reference events’ and how, and whether, these necessarily functioned as case examples of system-wide issues. Furthermore, there were challenges in extrapolating insights from case events to recommendations for the wider system. The introduction of a Scrutiny Panel and an enhanced role for the HSBI’s Intelligence Unit in identifying risks and selecting cases aimed to more systematically link ‘reference events’ with the potential for future system-wide learning. Such issues have been discussed with reference to the other forms of investigation and inquiry where retrospective learning from single events can be difficult to inform more proactice system-wide improvements.^3^ Echoing the recommendations of Hibbert et al.,^6^ health care safety investigation agencies need to dedicate considerable analytical attention to the identification and selection of events suitable for investigation, and particularly to how a reference event is illustrative of, and can provide the basis for, investigating and addressing broader system risks. Such processes would benefit from making systematic use of alternative information sources, especially where care systems use linked data,^25^ as well as designing investigations that connect multiple reference cases in complementary ways, to enable both a deep and broad exploration of risk and to inform comparative system-wide learning.

Conducting rigorous and systematic investigations is the core function of an investigation agency. Confirming Walshe’s research,^3^ HSIB’s approach to designing and carrying out investigations in its founding years was challenged by a lack of standardisation and agreement on the use of established safety investigation methodologies. There are various safety investigation methods and frameworks, informed by different schools of thought in the field of ‘safety sciences’, and with variable levels of application to different sectors.^12^ For HSIB, this challenge seemed to arise, in part, from the diverse backgrounds and experiences of its workforce and the pioneering nature of conducting system-wide, learning oriented investigations in health care. This meant that there were no immediately applicable methods that could be used more widely, and investigation processes were typically configured to the preferences and experiences of the particular investigators involved. As noted elsewhere^14^ questions remain as to whether the diverse backgrounds of HSIB’s workforce reflected a more deep seated problem resulting in a limited potential to translate investigatory methods from one sector to another. Through the course of this study, there was early evidence of HSIB moving towards a standardised investigation framework and approach. A key lesson from HSIB’s early experience is that considerable attention needs to be dedicated to the development and adaptation of investigation methodologies at a very early stage of an investigation agency’s configuration. Further, health care investigation bodies should work towards the development of a portfolio of broadly agreed methods and collaboratively and openly create a methodological toolkit that holds validity and credibility across the wider sector.^13^

Safety investigators, and the teams that support them, are the foundation of any safety investigation body and the principles of investigatory expertise must be embodied by investigators, and recognised by stakeholders across the health care system.^10,13^ HSIB’s workforce brought together people with experience of patient safety investigation within the NHS and those with investigative experience in other sectors, including transport and the military. This is in contrast to other sectors, where deep practical expertise of the sector is highly valued, and often a pre-requisite. This raises questions about knowledge and experience of the health care sector as an important feature of investigatory work. Bringing together staff with health care expertise and safety specialists from other sectors has the potential to foster cross-fertilisation of experience.^10^ How these different domains of expertise are integrated, how investigation teams are created and coordinated, and how staff with little experience of health care can be supported in developing sufficient knowledge of a diverse and complex sector, are key organisational challenges for new agencies like HSIB. However, we found that the absence of a centrally agreed and coordinated approach to investigation, and the diversity of the workforce contributed to the lack of standardisation in investigation processes and, more significantly, resulted in antagonism between groups. Much effort was invested in the early years to resolve these tensions and to determine to what extent HSIB should replicate the types of investigation agency found in other sectors or whether they should develop a distinct approach.^12^ This points to the critical importance of appropriate recruitment, induction and training of staff within health care safety investigation agencies, including a clear agreement on the competencies, skills and expertise that are required, and also looking beyond individual skills and competences to develop and coordinate the collective and combined talents of an investigation agency’s workforce.

Following completion of the study, and through ongoing engagement between HSIB and the University research team, we wish to highlight ongoing changes in HSIBs organisation and governance, much of which was triggered by the King’s Fund report.^14^ With regard to independence, HSIB has developed a more balanced approach between organisational freedom to decide upon its investigation work together with a renewed commitment to working with stakeholders in translating and implementing its learning. It has also developed a new framework for the selection of investigations that is moving away from ‘reference events’ to one informed by multiple sources of insight and intelligence around the most significant patient safety concerns.^6^ There has also been considerable work in developing a standardised investigation process with regular reviews and enhanced quality assurance, with greater attention given to the knowledge and skills of investigators according to the needs of the investigation. One of HSIB’s strengths is the diversity and multidisciplinary expertise in the investigation team. As an organisation they continue to ensure effective team working and maximise the opportunity to draw on the diverse skills of the workforce. These developments have culminated in a new operating framework and forward planning to match expertise in the team with specific investigations.

In thinking about the broader learning derived from HSIB’s early development, it could be argued that rather than seeking to learn from the examples of investigation agencies found in other safety-critical sectors, those involved in shaping and leading HSIB might have attended to the learning derived from other forms of investigation, inquiry and regulatory activity in the health care sector, which reveal many common challenges. It also questions the attraction of continually seeking to emulate or ‘copy-and-paste’ safety improvement methodologies that are designed for entirely different contexts.^26,27^

While our research provides important new insights, we acknowledge the limitations of our work. Firstly, as a qualitative study based on participant interviews, we did not have the opportunity to develop a fine-grained case study, for example using ethnographic research to examine individual investigations. Secondly, we did not track individual investigations to more closely examine their impact. An extremely important factor to consider is the highly charged political context at the time of our study, with much public debate and scrutiny about HSIB and the organisational culture and development.^28^ This contextual sensitivity likely shaped data collection, interview participant responses and the reporting of our study. However, this work paves the way for further study into investigatory approaches, reporting mechanisms and the impact of recommendations across the health care system. Furthermore, there is an opportunity to develop comparative and longitudinal research to explore our findings in different contexts.

## Conclusions

This study draws upon the unique experiences of HSIB to offer practical learning to health care decision-makers about the importance of securing independence and integration, about the production of system-wide learning, the standardisation of robust methodologies and the support for a multidisciplinary specialist workforce.
